# Simulation and Prediction of the Potential Distribution of *Spodoptera frugiperda* Under Current and Three Future Scenarios Based on the Biomod2 Model Under Global Climate Change

**DOI:** 10.3390/insects17080772

**Published:** 2026-07-27

**Authors:** Zhipeng He, Boyang Zhang, Rulin Wang, Danping Xu, Xinqi Deng, Zhihang Zhuo

**Affiliations:** 1 College of Life Science, China West Normal University, Nanchong 637002, China; 2 School of Architecture and Art, Hebei University of Architecture, Zhangjiakou 075000, China; 3 Sichuan Province Agro-Meteorological Center, Chengdu 610071, China; 4 Environment-Friendly and Efficient Water-Saving Technology and Equipment for Hilly Agriculture Key Laboratory of Sichuan Province, Chengdu 610066, China; 5 Environment and Plant Protection Institute, Chinese Academy of Tropical Agricultural Sciences, Haikou 571101, China

**Keywords:** *Spodoptera frugiperda*, climate change, biomod2, ArcGis, agricultural pests, ensemble model

## Abstract

*Spodoptera frugiperda* is a highly polyphagous pest that poses a serious threat to global agricultural production. Understanding its species distribution is of great importance under the current context of climate change. In this study, we used the biomod2 platform to integrate six models, namely XGBOOST, Maxnet, Maxent, MARS, GBM, and GLM, to construct an ensemble model. This ensemble model was used to simulate the potential distribution of *S. frugiperda* under current and three future climate scenarios, and to analyze the main environmental factors influencing its distribution and their change trends. The results indicated that Bio08, Bio16, Bio09, and Bio03 were the major environmental factors affecting the species’ distribution. Under the current scenario, the suitable area was approximately 2026.05 × 10^4^ km^2^, accounting for about 10% of the global land area, and was mainly concentrated in East Asia and central North America. The expansion of *S. frugiperda* varied among the three future climate scenarios; under the SSP5-8.5 scenario, the expansion toward higher latitudes was the most pronounced, with the total suitable area increasing by 91.8%. Analysis of the three climate scenarios revealed that the expansion trend of *S. frugiperda* intensified with increasing carbon emissions. Under the most conservative SSP1-2.6 scenario, even a slight reduction in suitable area was possible. This study provides an in-depth exploration of the distribution characteristics of *S. frugiperda* and its responses to environmental factors from a geographical perspective. These findings contribute to a better understanding of the species’ potential distribution and the influence of environmental variables from a spatial standpoint. They offer a scientific basis for the prevention and control of this pest in specific regions, and may also serve as a reference for future studies on similar invasive insect pests.

## Simple Summary

The fall armyworm is a pest that feeds on more than 350 plant species and causes severe damage to crops such as maize, cotton, and rice worldwide. Understanding where this insect can survive and spread is important for protecting food production. In this study, we used computer models to predict the potential living areas of the fall armyworm under current and future climate conditions. Our results show that the pest currently inhabits about 10% of the global land, mainly in East Asia and central North America. Four climate factors—temperature and rainfall conditions during the wettest and driest seasons—were found to be the main drivers of its distribution. Under future climate scenarios with higher carbon emissions, the suitable areas for this pest are projected to expand significantly toward higher latitudes, with the largest increase reaching nearly 92%. These findings provide valuable guidance for agricultural monitoring and pest control strategies in regions at risk of invasion.

## 1. Introduction

Climate change has become a significant threat to global biodiversity and the stability of agricultural and forestry ecosystems. In recent decades, rising temperatures, shifting precipitation patterns, and the increased frequency of extreme climate events have been profoundly reshaping the geographic distribution patterns and population dynamics of insect species [1,2]. Extreme climatic events induced by climate change have a more pronounced impact on the migration patterns of various agricultural pests, such as hemipteran insects and moths [3,4]. For example, a significant increase in the population of Spodoptera litura (Lepidoptera: Noctuidae) was observed in specific regions of Japan following typhoon events [5]. Climate change is profoundly altering the geographic distribution patterns of agricultural pests and directly regulating their suitable habitats. However, the responses of different pest species to climate change are not uniform, and thus further in-depth studies are still needed on a species-specific basis.

*Spodoptera frugiperda* (also known as the fall armyworm) (Lepidoptera: Noctuidae) is native to tropical and subtropical regions of the Americas and is a global agricultural pest [6]. *S. frugiperda* is a highly polyphagous pest that feeds on more than 353 plant species across 76 families, with its primary host plants being maize, cotton, sorghum, and rice [7]. *S. frugiperda* is a major agricultural pest belonging to the family Noctuidae (Lepidoptera). Its larval stage causes severe damage, with a remarkably high feeding capacity. The larvae can bore into the basal parts of plant seedlings, causing seedling death; under severe infestation, they may completely defoliate entire plants, leading to total crop failure [8,9]. Native populations of *S. frugiperda* are characterized by strong seasonal migration, particularly in the Americas, where populations expand northward during the growing season and retreat to warmer overwintering regions [10]. These migration dynamics have contributed to the species’ rapid spread and successful establishment following its invasion into Africa and other regions [11]. Since its first report in West Africa in 2016, this invasive species has rapidly expanded its distribution range, spreading across Africa, Asia, and Oceania, and has recently extended into Europe [11,12,13]. Current research on *S. frugiperda* has been fairly extensive, with studies mainly focusing on control measures, biological characteristics, and host plants [14,15,16]. However, research on species distribution remains relatively scarce [17]. This does not mean that species distribution studies are unimportant for this species; on the contrary, investigating species distribution not only helps to understand its climatic and environmental adaptability at a macro level, but also provides reliable scientific evidence for geographical monitoring [18].

Species distribution models (SDMs), also known as ecological niche models (ENMs), operate within a multidimensional ecological space defined by environmental variables. Based on statistical information derived from occurrence records, these models estimate the ecological niche requirements of a species and then project the results onto selected spatiotemporal scales, reflecting the species’ habitat preference in the form of probabilities. SDMs combine distribution data of the target species with environmental variables at sampling sites to fit the relationship between the species and its environment, thereby predicting the potential distribution areas of the species [19,20,21].

Commonly used base models include three regression techniques—Generalized Linear Model (GLM), Generalized Additive Model (GAM), and Multivariate Adaptive Regression Splines (MARS); five machine learning methods—Random Forest (RF), eXtreme Gradient Boosting (XGBOOST), Gradient Boosting Machine (GBM), Artificial Neural Network (ANN), and Classification Tree Analysis (CTA); one discriminant classification model—Flexible Discriminant Analysis (FDA); one range envelope method—Surface Range Envelope (SRE); and two maximum entropy models—Maximum Entropy (Maxent) and Maximum Entropy Network (Maxnet) [20,21,22,23].

In ecological niche modeling or general predictive modeling, although a single model is convenient to operate, it has certain limitations. Due to algorithmic bias, sensitivity to data, susceptibility to overfitting, and high uncertainty during extrapolation, a single model cannot accurately reflect the relationships between species and their environment, and its predictions are unreliable [24]. The biomod2 modeling platform was developed in 2003 and has been widely recognized in this field [25,26]. This platform enables the integration of the aforementioned 12 modeling algorithms, allowing users to select different models and optimize prediction results by adjusting initial conditions, model types, parameters, and boundary settings.

Although a few species distribution studies on *S. frugiperda* have been conducted to date, these studies have more or less certain limitations. For instance, Huang Yanru et al. used an optimized MaxEnt model to study the species’ distribution only in Yunnan Province, China. However, for a global agricultural pest, using occurrence records from only a local region cannot accurately and effectively project the distribution onto a large scale, nor can the resulting suitability represent that of most other regions [27]; In terms of large-scale studies, Muhammad N. Baloch et al. and Marian Adan et al. investigated the potential distribution of *S. frugiperda* across broad geographic regions using the Maxent algorithm. Specifically, Adan et al. developed a model based on occurrence records from Africa to evaluate the distribution of this species in Africa, whereas Baloch et al. used global occurrence records obtained from GBIF (http://www.gbif.org/, accessed date: 20 July 2026) to predict its potential distribution. Although these studies provided valuable insights into the geographic suitability of *S. frugiperda*, predictions based on individual algorithms may still be influenced by algorithm-specific assumptions and uncertainties [13,28]. Therefore, this study complements previous research by applying an ensemble modeling framework that integrates multiple tuned algorithms, a commonly recommended approach for improving the robustness of species distribution models [29]. Therefore, based on the distribution data and environmental variables of *S. frugiperda*, this study used the Biomod2 modeling platform to integrate 12 individual models for simulation, and subsequently selected the best-performing models to construct an ensemble model for predicting the potential suitable distribution areas of *S. frugiperda*. The study aims to identify the optimal model for *S. frugiperda*, determine the key environmental variables affecting its distribution, and analyze its responses to climate change, thereby providing a scientific basis for future precise prevention and control of this pest.

## 2. Materials and Methods

### 2.1. Sources of Occurrence Data

**Figure 1:**
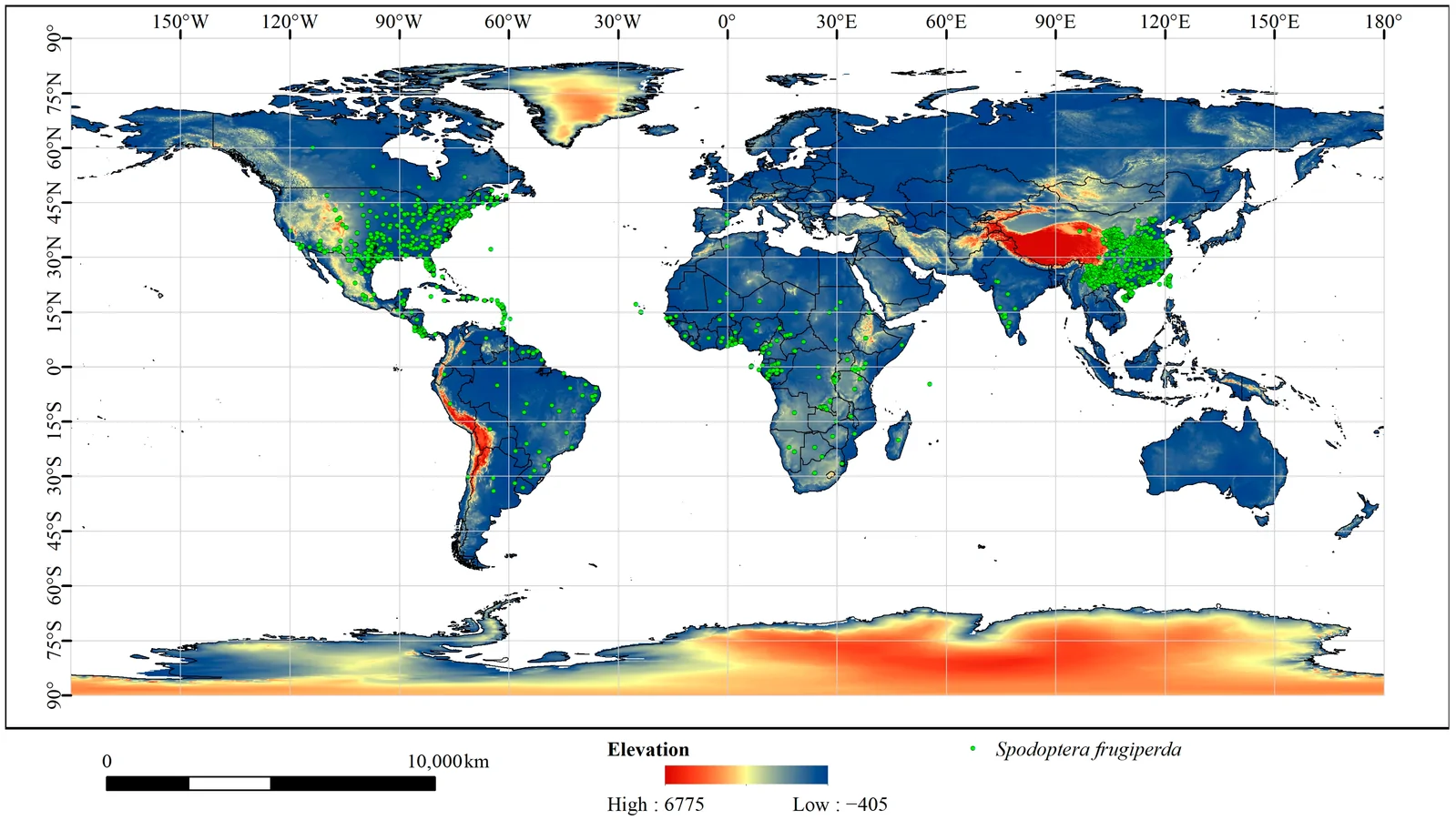
Geographic distribution records of *Spodoptera frugiperda*. The color gradient from blue to red represents elevation from low to high, and green dots indicate the recorded occurrence points.

The distribution data of *S. frugiperda* used in this study were obtained from multiple sources, primarily from the Global Biodiversity Information Facility (GBIF, http://www.gbif.org/). Additional data were derived from literature retrieved through academic databases, with specific latitude and longitude coordinates determined with the assistance of Google Earth (http://www.earthol.com/). Furthermore, some records were obtained through field surveys. All collected records were screened to remove non-natural occurrence points (e.g., introduced populations) and duplicate data. To reduce spatial clustering bias, the occurrence data were processed using ENMTools, retaining only one occurrence point within each 2.5 km grid cell. Ultimately, 1763 valid occurrence points were obtained after screening (Figure 1).

### 2.2. Environmental Data

Environmental factors are crucial for predicting and simulating species distributions. In this study, in addition to the 22 environmental variables commonly used for modeling the potential distribution of insects (19 climatic factors + 3 topographic factors; see Table S1), we incorporated vegetation coverage. The 23 environmental variables were derived from the WorldClim 2.1 climate database (https://worldclim.org/) [30], while vegetation coverage data were obtained from GlobalMaps (https://globalmaps.jp/). Future climate data were sourced from the ScenarioMIP simulations under the framework of the Sixth Coupled Model Intercomparison Project (CMIP6), using the BCC-CSM2-MR global climate model. Data covered three Shared Socioeconomic Pathway scenarios (SSP1-2.6, SSP2-4.5, and SSP5-8.5) across three time periods: the 2050s (2041–2060), 2070s (2061–2080), and 2090s (2081–2100).

When predicting the potential distribution of *S. frugiperda*, the climatic and environmental variables may exhibit strong autocorrelation, and not all variables are necessarily required for prediction. To avoid overfitting caused by multicollinearity among environmental factors, this study used Pearson correlation coefficients (r) to diagnose multicollinearity among environmental variables (see Figure S1). Based on modeling results and ecological interpretability, variables with absolute correlation coefficients greater than 0.8 were excluded to reduce the impact of multicollinearity on model predictions. Ultimately, 13 environmental variables were selected for subsequent modeling (Table 1).

### 2.3. Model Construction and Optimization

Model construction was primarily based on the biomod2 platform. Since the suitability of each model for predicting the distribution of *S. frugiperda* was unknown, we first trained all 12 models individually, selected the best-performing models, and then constructed an ensemble model on the biomod2 platform using a weighted average approach. Building the ensemble model required species distribution data and pseudo-absence data. The platform provides several methods for generating pseudo-absence points based on background data. In this study, we used the “random” command to generate 10,000 pseudo-absence points for model simulation [31]. In addition, we used the “biomodtuning” command for parameter optimization and split the dataset into a 75% training set and a 25% validation set. The occurrence points and pseudo-absence points were assigned equal weights, and the procedure was repeated 10 times to improve the stability of the results [32].

Model prediction accuracy was evaluated using the Receiver Operating Characteristic curve (ROC), the True Skill Statistic (TSS), and Cohen’s Kappa coefficient (Kappa), which measures the agreement between predicted and observed classifications beyond chance, and the model outputs were calibrated and validated [33]. Models with ROC > 0.9 and TSS > 0.7 were selected as high-quality models for building the ensemble model. Based on the threshold derived from the ensemble model, the study area was classified into unsuitable and suitable zones; the suitable zones were further subdivided into three classes: low, moderate, and high suitability.

### 2.4. Suitable Area Classification and Centroid Shift

The Biomod2 model results were evaluated using TSS, ROC, and Kappa scores to select the optimal model. After constructing the ensemble model, it was projected onto future environmental variables to predict the potential geographic distribution. The resulting distribution maps were normalized in ArcGIS 10.8. Based on the Biomod2 outputs, areas with values above the threshold (218) were designated as suitable areas, which were further classified into low-suitability (218–430), moderate-suitability (430–642), and high-suitability (642–854) zones based on equal intervals [34]. Subsequently, the Raster Calculator tool was used to reclassify data under different climate scenarios; meanwhile, centroid analysis was performed using the SDMsTools in ArcGIS (version 10.8) to calculate and visualize the distribution centroids across different scenarios.

## 3. Results

### 3.1. Model Accuracy

The running results indicated that all 12 models were successfully executed, and model parameters were optimized using the “biomod_tuning” function. The parameters of each iteration were evaluated based on ROC, Kappa, and TSS, and the optimized results achieved relatively high accuracy. Among all successfully run models, RF achieved ROC, Kappa, and TSS values of 1.0, indicating clear overfitting; XGBOOST (version 3.4.0), Maxent (version 3.4.4), and Maxnet exhibited superior performance, with mean TSS values above 0.75 and mean ROC values above 0.94; GBM, GLM, and MARS showed good performance, with mean TSS values above 0.7 and mean ROC values above 0.9; CTA, GAM, FDA, and SRE demonstrated moderate performance; while ANN performed extremely poorly, with TSS values below 0.5 and ROC values below 0.7 (Table 2).

RF was excluded from the construction of the ensemble model as a priority due to its risk of overfitting and potentially weak generalization ability. Six models with excellent and good performance were selected and integrated using a weighted averaging approach to construct the final ensemble model. The ensemble model exhibited excellent performance, with a TSS value of 0.851 and an ROC value of 0.977, providing a reliable basis for the subsequent simulation results.

### 3.2. Key Environmental Variables Used in Model Construction and Their Response Curves

**Figure 2:**
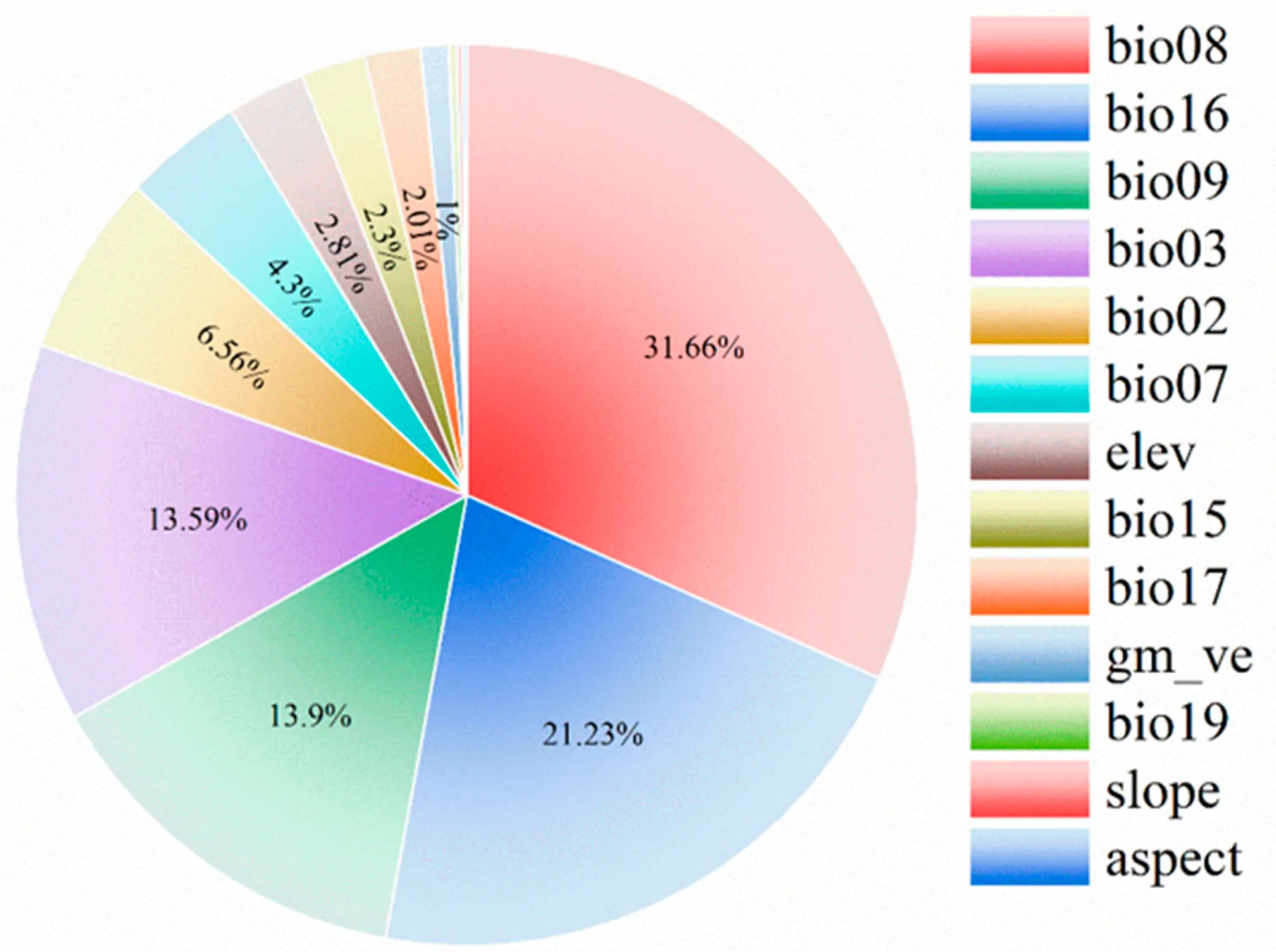
Importance of environmental variables used in model construction.

Through the biomod2 platform, this study evaluated the contribution of each environmental variable in the modeling process. The results showed that different models adopted different combinations of variables, with substantial variation in the contribution of each variable across models. Based on the best-performing ensemble model, we further analyzed the relative importance of environmental variables influencing the distribution of *S. frugiperda*. Among the 13 variables selected for modeling, 10 had importance values exceeding 1%, while Bio08, Bio16, Bio09, and Bio03 each exceeded 10% and were identified as key environmental variables. The cumulative importance of these four variables reached 80.4%, with Bio08 alone accounting for the highest proportion at 31.66% (Figure 2).

**Figure 3:**
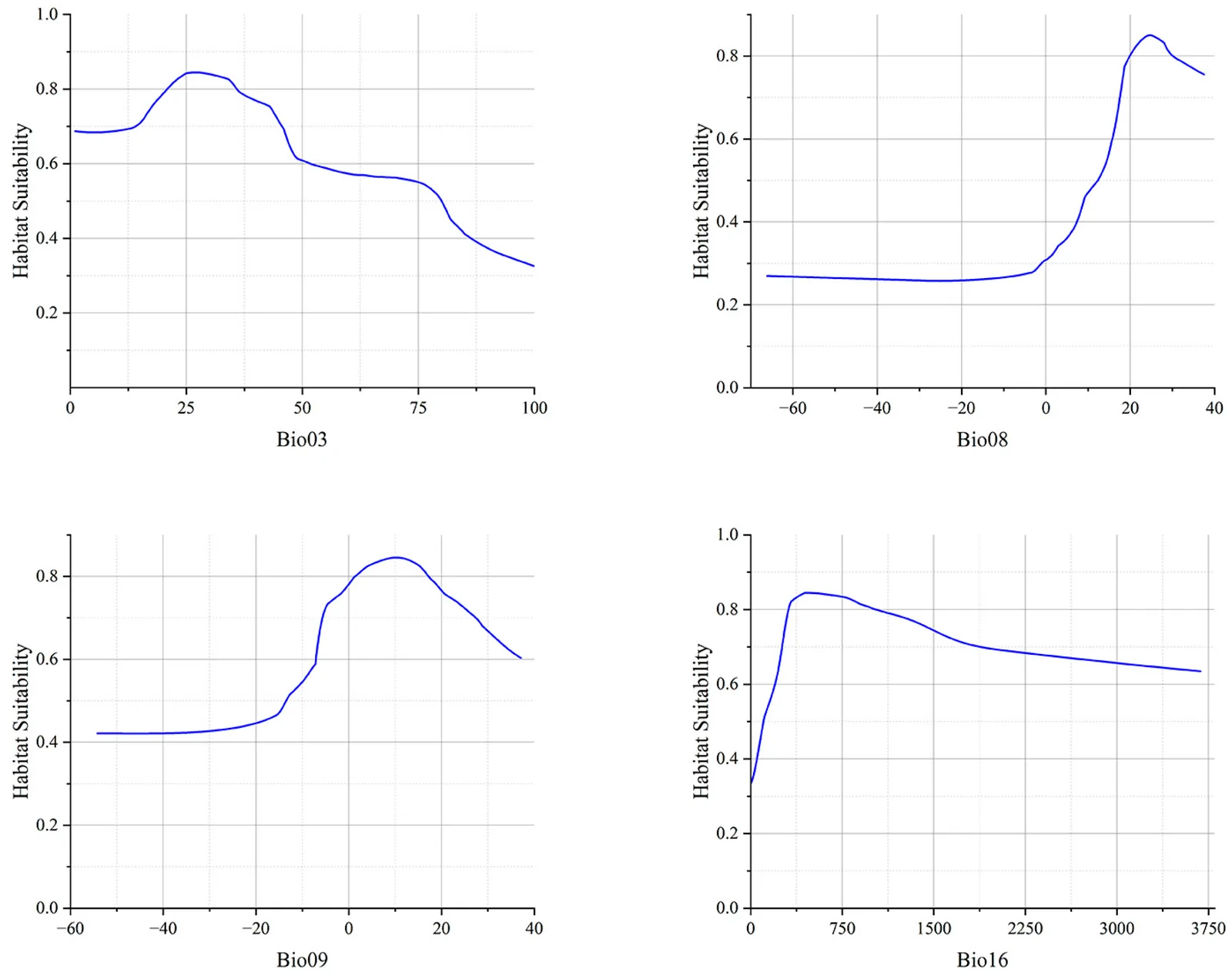
Response curves of the occurrence probability of *S. frugiperda* to key environmental variables.

Based on the response data of key environmental variables output from the biomod2 platform, this study generated response curves illustrating the relationship between the occurrence probability of *S. frugiperda* and the four key variables: Bio03, Bio08, Bio09, and Bio16 (Figure 3). The response curve of Bio03 exhibited an initial increase followed by a decrease, with suitability occurring within the range of 0–51.80 °C and peaking between 25.02% and 28.05%. The response curve of Bio08 showed a similar unimodal pattern, with suitability within 15.78–37.40 °C and a peak between 24.38 and 24.86 °C. The response curve of Bio09 also displayed an initial rise and subsequent decline, with suitability within −7.04–37.14 °C and a peak between 9.20 and 11.08 °C. The response curve of Bio16 followed the same trend, with suitability within 197.33–3684.74 mm and a peak between 437.12 and 457.92 mm.

### 3.3. Potential Distribution of Spodoptera frugiperda Under the Current Climate Scenario

**Figure 4:**
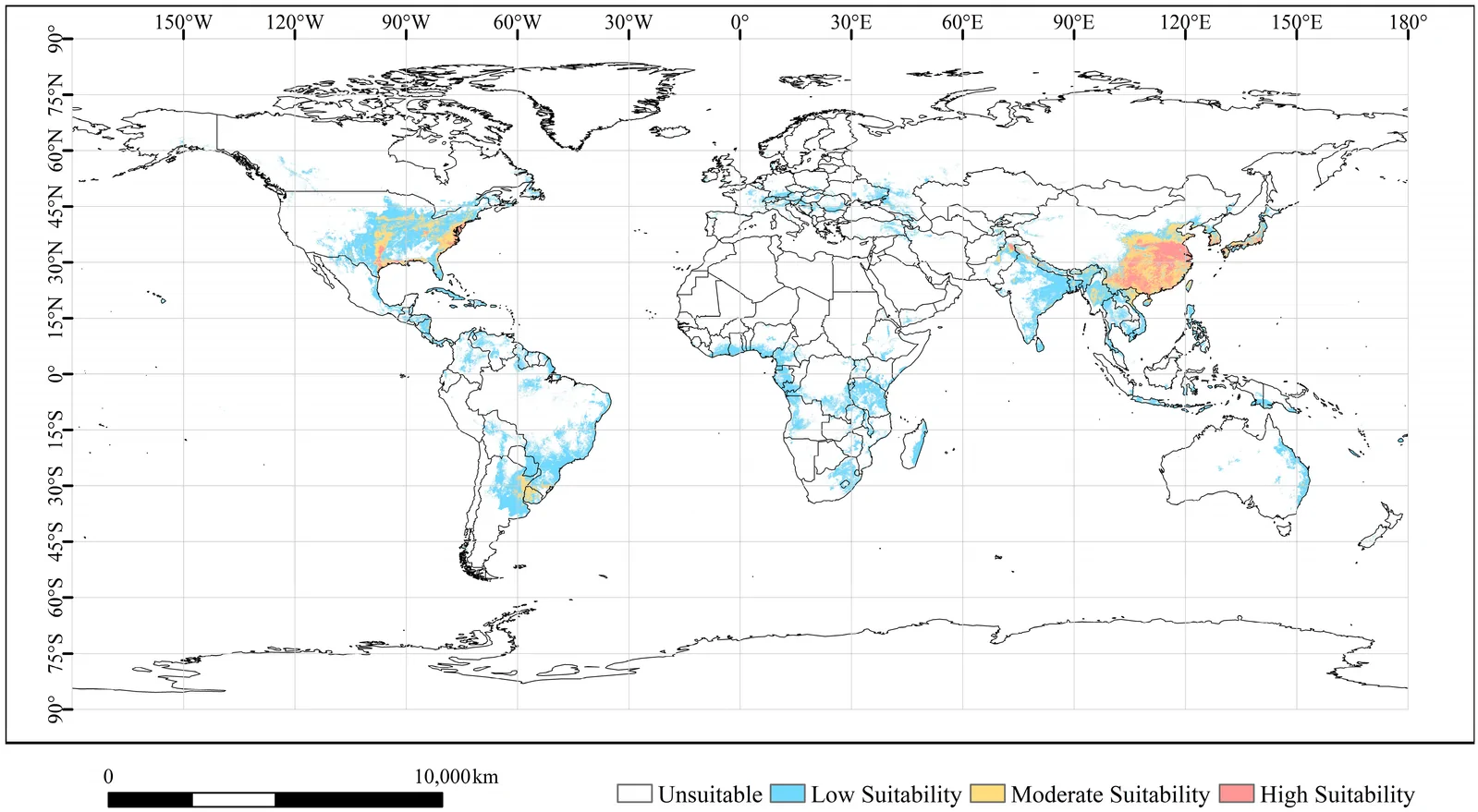
Potential suitable areas for *S. frugiperda* under the current scenario (white: unsuitable; blue: low suitability; yellow: moderate suitability; red: high suitability).

According to the ensemble model predictions, under the current climate scenario, approximately 2026.05 × 10^4^ km^2^ of the global land area was identified as potentially suitable for *S. frugiperda*. Among this, the high-suitability area covered about 147.63 × 10^4^ km^2^, accounting for 7.29% of the total suitable area, and was mainly distributed in eastern Asia, with a smaller portion in south-central North America. The moderate-suitability area covered approximately 338.73 × 10^4^ km^2^, representing 16.72% of the total suitable area, and was also predominantly located in eastern Asia, with smaller areas in south-central North America and scattered occurrences in East Asia, Southeast Asia, and southern South America. The low-suitability area covered about 1539.68 × 10^4^ km^2^, comprising 75.99% of the total suitable area. In addition to overlapping with the moderate-suitability zones, the low-suitability areas extended to western and southern Europe, eastern Australia, and central Africa (Figure 4). Overall, the suitable areas for *S. frugiperda* occupied nearly 10% of the global land surface, with the main distribution concentrated between 45° N and 45° S.

### 3.4. Potential Distribution of Spodoptera frugiperda Under Multiple Future Climate Scenarios

**Figure 5:**
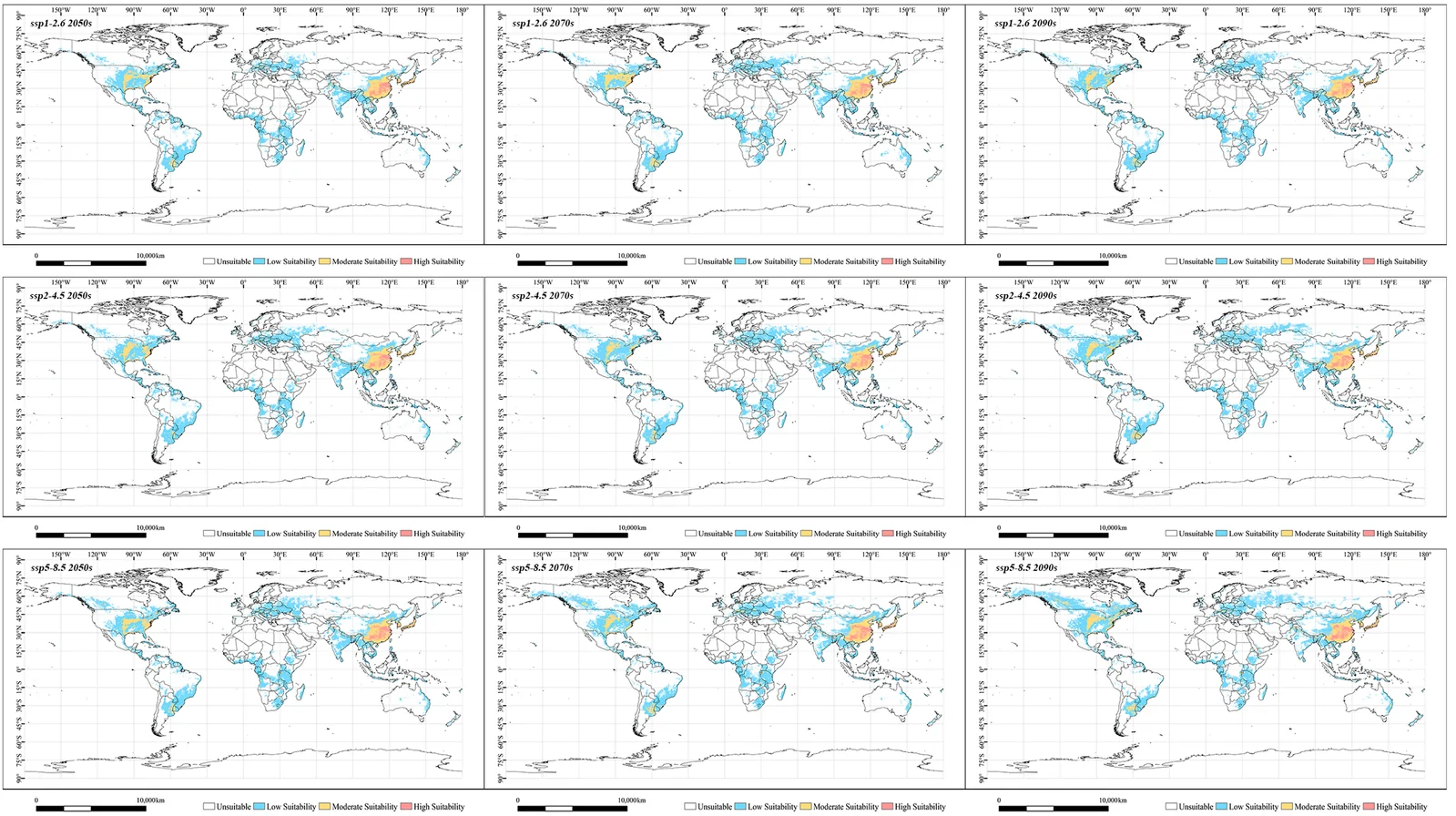
Potential suitable areas of *S. frugiperda* under three future scenarios (Top row: SSP1-2.6 scenario for the 2050s, 2070s, and 2090s; middle row: SSP2-4.5 scenario for the 2050s, 2070s, and 2090s; bottom row: SSP5-8.5 scenario for the 2050s, 2070s, and 2090s.).

The model successfully simulated the potential distribution under three future scenarios across three time periods. Overall, the changes in the potential distribution of *S. frugiperda* under future scenarios were not substantial, with the core suitable areas still concentrated in East Asia, Southeast Asia, south-central North America, central Europe, southeastern South America, and south-central Africa (Figure 5). Under the SSP1-2.6 scenario, the most noticeable changes occurred in central Europe, showing a clear northward expansion; other changes mainly involved outward radiation from high-suitability areas and poleward expansion of low-suitability areas. The change pattern under the SSP2-4.5 scenario was similar to that under SSP1-2.6, but the outward expansion of areas of low suitability was slightly stronger. Under the SSP5-8.5 scenario, the changes were primarily reflected in the westward expansion of the core suitable area in south-central North America, with a very pronounced magnitude; by the 2070s and 2090s, low suitability had already expanded into southern North America (Figure 5).

In terms of area changes, under the SSP1-2.6 scenario, the low suitability of *S. frugiperda* showed a continuous increasing trend, while the moderate suitability increased first and then decreased, and the high-suitability areas exhibited a slight decrease. Nevertheless, the overall potential distribution still showed an expanding trend, with the total suitable areas accounting for 14.22%, 14.61%, and 14.84% of the global land area in the three time periods, respectively. Under the SSP2-4.5 scenario, the low-suitability areas continued to increase, the moderate-suitability areas decreased first and then increased, and the high-suitability areas showed a slight increase, with the overall distribution also presenting an increasing trend; the proportions were 15.03%, 15.14%, and 16.17%, respectively. Under the SSP5-8.5 scenario, all three suitability classes showed sustained increasing trends, with the suitable areas accounting for 15.76%, 17.02%, and 18.12% of the global land area in the three periods, respectively (Table 3).

### 3.5. Simulation of Distribution Changes and Centroid Shifts in S. frugiperda

**Figure 6:**
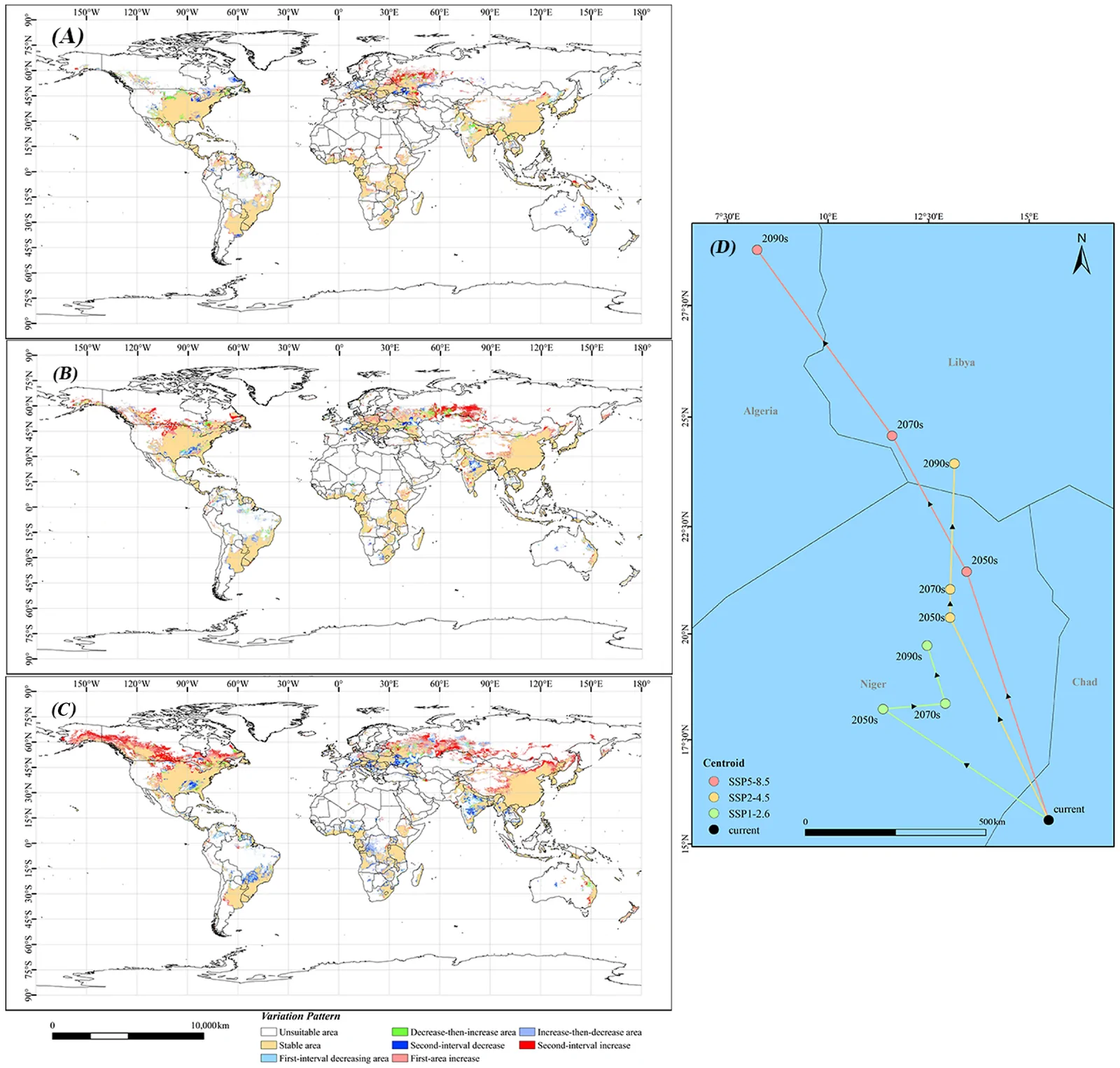
Spatial changes and centroid shifts in *S. frugiperda* under three future scenarios. (**A**) SSP1-2.6; (**B**) SSP2-4.5; (**C**) SSP5-8.5; (**D**) Centroid displacement map.

After sorting and analyzing the model outputs under different climate scenarios, the following distributional trends were observed. Under the SSP1-2.6 scenario, the most pronounced changes occurred in central Europe, showing an eastward expansion trend, while eastern Australia exhibited a relatively clear westward contraction. Changes in East Asia, South Africa, southern South America, and central North America were less evident, with only slight reductions in suitable areas (Figure 6A). Under the SSP2-4.5 scenario, central Europe showed a more obvious eastward expansion, and central North America also exhibited a westward expansion trend, whereas East Asia, South Africa, Australia, and southern South America displayed relatively moderate changes (Figure 6B). Under the SSP5-8.5 scenario, south-central North America showed a marked westward expansion, accompanied by a notable contraction of suitable areas within the region; central Europe exhibited a clear eastward spread; changes in East Asia, South Africa, Australia, and southern South America remained relatively moderate (Figure 6C).

The model data were processed using the SDMsTools in ArcGIS (version 10.8) to visualize the distribution centroids under different scenarios (Figure 6D). The centroid analysis was conducted at the global scale, and the resulting centroid shifts represent changes in the geographic center of climatically suitable habitats under different climate scenarios. The results showed a clear northward shift in the centroids over time under all climate scenarios. Under the current scenario, the centroid of *S. frugiperda* was located at 15.489° E, 15.573° N, in Chad. Under the SSP1-2.6 scenario, the centroid displacement was relatively small; over the three time periods, the centroid first moved 528.9 km northwestward, then 164.5 km northeastward, and finally 160.2 km northwestward, reaching 12.462° E, 19.729° N. All three centroids under this scenario were located in Niger. Under the SSP2-4.5 scenario, the displacement was more pronounced; the centroid first moved 594.7 km northwestward, then 73.5 km northeastward, and finally 322.4 km northeastward, reaching 13.142° E, 23.943° N. Except for the centroid in the 2090s, which fell in Libya, all other centroids under this scenario were located in Niger. Under the SSP5-8.5 scenario, the displacement was the most substantial; the centroid first moved 688.5 km northwestward, then 395.7 km northeastward, and finally 569.4 km northwestward, reaching 8.234° E, 28.726° N. The three centroids under this scenario were located in Niger, Libya, and Algeria, respectively.

## 4. Discussion

Compared with plants, using climatic factors to predict the species distribution of animals generally yields better results. This is mainly attributed to the fact that animals have a strong capacity for active dispersal, whereas plants lack the ability for autonomous movement. The latter may lead to difficulties in distinguishing naturally distributed wild individuals from artificially cultivated ones in actual sampling, thereby introducing data bias [35]. For animals, climatic factors show significant advantages in predicting the species distribution of small-bodied species [22]. For *S. frugiperda*, an invasive species with virtually no artificial rearing, the interference of anthropogenic factors in distribution data is further reduced, thereby making the prediction results more robust [36]. In addition, to compensate for the potential algorithmic biases and uncertainties inherent in single-model approaches, this study employed the Biomod2 ensemble modeling framework to integrate predictions from multiple algorithms, providing a more reliable foundation for subsequent analyses. During model construction, after rigorous screening, six models—XGBOOST, Maxnet, Maxent, MARS, GBM, and GLM—were selected for ensemble modeling in this study, covering three types of algorithms: regression models, machine learning models, and maximum entropy models. The ensemble model demonstrated high reliability, with evaluation metrics reaching a TSS value of 0.851 and an ROC value of 0.977, both outperforming any individual model, thereby providing a solid foundation for subsequent analyses.

In model construction, considering that *S. frugiperda* is a phytophagous pest, we included a vegetation coverage factor (gm_ve). However, this variable did not perform notably well in the models, possibly because general vegetation indices cannot accurately represent the spatial availability of preferred host plants. Although maize and other grasses are important hosts of *S. frugiperda*, developing a global-scale host distribution layer remains challenging due to its broad host range, seasonal crop changes, and the strong influence of agricultural management practices [7]. Future studies integrating dynamic crop distribution datasets and host availability information may further improve predictions of the realized distribution of this species [37]. In addition, four key environmental variables were identified: Bio08 (mean temperature of the wettest quarter), Bio16 (precipitation of the wettest quarter), Bio09 (mean temperature of the driest quarter), and Bio03 (isothermality). Among these, Bio08 reflects the thermal conditions during the growing season and is crucial for determining whether noctuid moths can successfully complete their life cycle. Its optimal range peaked at 24.4–24.9 °C, which falls within the optimal temperature window for the growth and development of most Lepidoptera (especially Noctuidae), a finding consistent with previous studies [38,39]. Bio16 (precipitation of the wettest quarter) reflects water availability during critical periods, directly influencing the growth of larval food plants and the humidity of the habitat [40]. Its suitable range is extremely broad (approximately 200 mm to 3685 mm), indicating that this species has a wide ecological amplitude with respect to precipitation, enabling it to survive under conditions ranging from semi-arid to humid climates. This finding is also corroborated by the results of Xu J.H. et al. [38]. Bio09 (mean temperature of the driest quarter) reflects the environmental stress faced by the noctuid moth during the dry season. Its suitable temperature range spans from −7.04 to 37.14 °C, suggesting that the occurrence records of *S. frugiperda* are associated with a broad range of Bio09 values, although this does not necessarily indicate physiological tolerance to extremely low temperatures. Previous studies have shown that temperatures below approximately 10 °C can negatively affect the survival and development of *S. frugiperda* [41]. The relatively broad response range observed in this study may be related to seasonal migration, local microclimatic conditions, or the influence of other environmental factors not included in the model. The optimal peak occurs at approximately 10 °C, suggesting a preference for relatively cool environments, a finding that is consistent with previous studies [42,43]. Bio03 (isothermality) represents the ratio between diurnal temperature variation and annual temperature variation. The response curve indicates that *S. frugiperda* shows higher suitability under relatively low Bio03 values, suggesting that areas with greater seasonal temperature variation relative to daily temperature variation may provide more suitable conditions for this species. This pattern is consistent with the results reported by Zhang X.Y. et al. [44]. Although this invasive species possesses a wide ecological amplitude, its distribution is likely determined by the combined effects of temperature, precipitation, seasonal dynamics, and other ecological processes.

From the perspective of suitable area distribution, *S. frugiperda* does show a clear preference for favorable climatic environments. Both the species occurrence records and the potential distribution maps indicate that its distribution is mainly concentrated in China and the United States. China and the United States are globally recognized as two major agricultural countries and are also the two largest maize producers in the world; consequently, *S. frugiperda* infestation is particularly severe in these regions [45]. In the simulation of future climate scenarios, we found that, under all scenarios, there was a notable change in suitable areas between the current scenario and the 2050s. However, in the subsequent two periods (i.e., 2050–2070s and 2070–2090s), the magnitude of change was substantially smaller than that from the current scenario to the 2050s. This can be attributed to the difference in time spans: the climate data for the current scenario are based on averages from 1970 to 2000, so the time span from the current scenario to the 2050s (approximately 50–80 years) is much larger than the 20-year intervals between future scenarios [30]. Therefore, by classifying and overlaying the potential distribution maps under different future climate scenarios (Figure 6A–C), it can be clearly observed that, under the broader context of climate change, the changes in suitable areas across the three future scenarios exhibit a distinct gradient response pattern. Under the SSP1-2.6 scenario, the high-suitability areas showed a slow decrease, while the Low Suitabilitys expanded northward, indicating that moderate warming may extend marginal suitable areas but could reduce the suitability of some core regions. The change trend under the SSP2-4.5 scenario was similar to that of SSP1-2.6, but all suitability classes showed an overall increase, albeit with relatively modest magnitudes, possibly reflecting that under a medium-emission pathway, the promoting effects of rising temperatures begin to outweigh the inhibiting effects. Under the SSP5-8.5 scenario, the increase in suitable area was the most pronounced (with a total increase rate of 91.8%), with all three suitability classes showing continuous expansion. Spatially, the low suitability in North America and Europe expanded markedly northward and westward. The primary reason for this gradient response is that the SSP5-8.5 scenario results in the most severe global warming due to greenhouse gas emissions, which substantially reduces the constraints of winter low temperatures on the survival and overwintering of *S. frugiperda* in mid- and high-latitude regions, while also extending the effective accumulated temperature period during the growing season, thereby facilitating the species’ expansion into previously unsuitable higher-latitude areas [46].

It should be noted that the prediction results of this study can only provide a qualitative analysis of the potential distribution of *S. frugiperda*, rather than quantitative conclusions. Moreover, discrepancies between predicted suitability and reported pest severity in some regions (e.g., Indonesia, the Philippines, and northeastern Australia) may result from incomplete occurrence records and the inability of SDMs to fully incorporate agricultural practices, host crop distribution, and migration dynamics [29,47]. Therefore, predicted suitability should be interpreted as climatic potential rather than actual infestation intensity [37]. Species distribution models—whether based on a single algorithm or the Biomod2 ensemble framework—commonly share several inherent limitations. First, models are essentially simplified approximations of real ecological processes and cannot account for adaptive evolution or physiological plasticity responses that species may undergo under long-term climate change [48]. Second, models are primarily based on abiotic environmental factors (e.g., temperature and precipitation) and fail to incorporate biotic interactions such as competition, predation, and parasitism, which profoundly influence species distribution patterns in practice [49]. Future studies could incorporate population dynamics models such as DYMEX to better simulate population processes, while climate-based approaches such as CLIMEX may provide additional insights into climatic suitability under changing environments [50]. In addition to the aforementioned common limitations of SDMs, this study also has scale-related limitations. Although global-scale predictions are helpful for understanding the overall distribution pattern, different geographical populations of *S. frugiperda* may exhibit niche differentiation due to local adaptation, and their suitable conditions may vary regionally; such specificity is difficult to capture fully in the global-scale simulations of this study [7]. Moreover, the realized distribution of *S. frugiperda* is likely influenced not only by climatic suitability but also by ecological processes that were not explicitly incorporated into the present models. As a highly migratory pest, its annual distribution may be shaped by seasonal migration and temporal variation in host crop availability [10,47]. In addition, climate change may affect migration timing, overwintering boundaries, and crop phenology, thereby further influencing future distribution patterns [51]. Integrating these ecological processes into future SDMs may improve the predictive accuracy of species distribution under changing environmental conditions [37]. Future research should combine regional-scale climate data with local population physiological experiments to further refine the suitability assessments for different geographical populations.

## 5. Conclusions

In this study, we used the biomod2 platform to simulate and predict the global distribution of *S. frugiperda* by employing 12 species distribution models in combination with 22 environmental variables, and subsequently constructed an ensemble model based on the best-performing individual models. The ensemble model exhibited superior predictive performance compared to any single model, with accuracy metrics of TSS = 0.851 and ROC = 0.977. The four most important environmental variables in the modeling process were Bio08 (31.66%), Bio16 (21.23%), Bio09 (13.90%), and Bio03 (13.59%). Under the current climate scenario, the suitable areas for *S. frugiperda* are mainly distributed in East Asia, south-central North America, southern South America, and southern Africa, with a total suitable area of 2026.05 × 10^4^ km^2^, accounting for approximately 10% of the global land area. The ensemble model was successfully projected onto three time periods under three future representative carbon emission scenarios. The results indicate that over time, the suitable areas for *S. frugiperda* will continue to expand northward and westward, and this expansion trend will become more pronounced with increasing carbon emission levels. This study, from a geographical distribution perspective, aims to further clarify the potential distribution of *S. frugiperda* and the environmental factors affecting it, so as to provide a reference for the prevention and control of this pest in specific regions, and to serve as a basis for future research on similar invasive insect pests.

## Figures and Tables

**Table 1:** Environmental variables used in this study and their descriptions.

Abbreviation	Description
aspect	The direction or orientation of the Earth’s surface
bio02	Mean diurnal range (monthly mean (max temp − min temp)) (°C)
bio03	Isothermality (BIO2/BIO7) × 100
bio07	Temperature annual range (BIO5-BIO6) (°C)
bio08	Mean temperature of the wettest quarter (°C)
bio09	Mean temperature of the driest quarter (°C)
bio15	Precipitation seasonality (C of V)
bio16	Precipitation of the wettest quarter (mm)
bio17	Precipitation of the driest quarter (mm)
bio19	Precipitation of the coldest quarter (mm)
elev	Elevation of the terrain
gm_ve	Percentage of vegetation coverage
slope	Slope or obliquity of the terrain

**Table 2:** Mean evaluation metrics of individual model performance and evaluation metrics of the ensemble model (values rounded to three decimal places).

Name	KAPPA	TSS	ROC
RF	1.000	1.000	1.000
XGBOOST	0.824	0.858	0.976
Maxnet	0.758	0.786	0.957
Maxent	0.745	0.753	0.947
MARS	0.700	0.732	0.933
GBM	0.687	0.716	0.937
GLM	0.685	0.701	0.933
CAT	0.685	0.655	0.893
FAD	0.573	0.617	0.873
GAM	0.585	0.604	0.891
ANN	0.480	0.520	0.770
SRE	0.441	0.434	0.717
EM	0.728	0.851	0.977

**Table 3:** Suitable areas of *S. frugiperda* under current and future scenarios across three time periods (unit: ×10^4^ km^2^).

Scenario	Period	Low Suitability	Moderate Suitability	High Suitability
current		1539.68	338.73	147.63
SSP1-2.6	2041–2060	2486.94	459.84	103.22
	2061–2080	2542.23	498.95	93.65
	2081–2100	2639.49	450.09	93.49
SSP2-4.5	2041–2060	2683.32	442.42	98.87
	2061–2080	2716.32	426.37	104.79
	2081–2100	2911.17	454.39	103.52
SSP5-8.5	2041–2060	2810.99	458.45	111.66
	2061–2080	3069.59	467.42	115.48
	2081–2100	3244.57	518.33	123.78

## Data Availability

For manuscripts that are accepted, all authors agree to make data and materials available to third-party academic researchers upon reasonable request.

## Supplementary Materials

The following supporting information can be downloaded at: https://www.mdpi.com/article/10.3390/insects17080772/s1. Table S1. All environmental variables used in this study; Figure S1. Correlation heatmap of climatic variables.

## References

[1] Colares C., Roza A.S., Mermudes J.R.M., Silveira L.F.L., Khattar G., Mayhew P.J., Monteiro R.F., Nunes M.F.S.Q., Macedo M.V. (2021). Elevational specialization and the monitoring of the effects of climate change in insects: Beetles in a Brazilian rainforest mountain. Ecol. Indic..

[2] Kuczyk J., Müller C., Fischer K. (2021). Plant-mediated indirect effects of climate change on an insect herbivore. Basic Appl. Ecol..

[3] Otuka A., Huang S., Sanada-Morimura S., Matsumura M. (2012). Migration analysis of *Nilaparvata lugens* (Hemiptera: Delphacidae) from the Philippines to Taiwan under typhoon-induced windy conditions. Appl. Entomol. Zool..

[4] Torres J.A. (1992). Lepidoptera outbreaks in response to successional changes after the passage of hurricane hugo in Puerto Rico. J. Trop. Ecol..

[5] Murata M., Etoh T., Itoyama K., Tojo S. (1998). Sudden occurrence of the common cutworm, *Spodoptera litura* (Lepidoptera: Noctuidae) in southern Japan during the typhoon season. Appl. Entomol. Zool..

[6] Russo M.L., Jaber L.R., Scorsetti A.C., Vianna F., Cabello M.N., Pelizza S.A. (2021). Effect of entomopathogenic fungi introduced as corn endophytes on the development, reproduction, and food preference of the invasive fall armyworm *Spodoptera frugiperda*. J. Pest. Sci..

[7] Nam K., Gimenez S., An H., Durand K., Gasser M., Yainna S., Legeai F., Beuzelin J., Heckel D.G., Hänniger S. (2026). Retrotransposed gene copies persist under relaxed selection in wild *Spodoptera frugiperda*. Mol. Biol. Evol..

[8] Badhai S., Gupta A.K., Koiri B. (2020). Integrated management of fall armyworm (*Spodoptera frugiperda*) in maize crop. Rev. Food Agric. (RFNA).

[9] Wang J., Mu Y., Yang C., Huang X., Wang Y., Yu H., Chen X. (2022). Effects of high and low temperature stress on growth, development and cold tolerance of *Spodoptera frugiperda* (Smith) (Lepidoptera: Noctuidae). J. Mt. Agric. Biol..

[10] Johnson S.J. (1987). Migration and the life history strategy of the fall armyworm, *Spodoptera frugiperdaSpodoptera frugiperda* in the Western Hemisphere. Int. J. Trop. Insect Sci..

[11] Goergen G., Kumar P.L., Sankung S.B., Togola A., Tamò M. (2016). First report of outbreaks of the fall armyworm *Spodoptera frugiperda* (J E Smith) (Lepidoptera, Noctuidae), a new alien invasive pest in West and Central Africa. PLoS ONE.

[12] Qian L., Lu E., Liu J., Wu Q. (2025). Migration patterns of *Spodoptera frugiperda* in China from 2019 to 2023 and their response to typhoons. J. Plant Prot..

[13] Adan M., Tonnang H.E.Z., Greve K., Borgemeister C., Goergen G. (2025). Modelling the environmental and terrestrial drivers of the spread of the invasive fall armyworm *Spodoptera frugiperda* in Africa. Crop Prot..

[14] Hamida D., Mallaiah B., Ramya V., Sree K.V., Bhadru D., Pushpavalli S. (2026). Maize-derived endophytic bacillus subtilis induces lethal and sublethal effects in the fall armyworm, *Spodoptera frugiperda*. J. Sci. Res. Rep..

[15] Gudla K., Mookiah S., Marimuthu M., Kesharivarmen S.G., Sampathrajan V., Gnanadhas P., Govindasamy K., Kolanchi P. (2026). Metabolite-guided disruption of *Spodoptera frugiperda*: Molecular docking and EAD insights for targeted control. J. Plant Dis. Prot..

[16] Luo D., Yu B., Jian B., Hu M., Zhao Y. (2026). *ScbHLH5* forms a feedback regulatory loop with jasmonic acid (JA) signalling pathway to enhance resistance to fall armyworm (*Spodoptera frugiperda*) in sugarcane. Plant Cell Environ..

[17] Fan S., Chen C., Zhao Q., Wei J., Zhang H. (2020). Identifying potentially climatic suitability areas for *Arma custos* (Hemiptera: Pentatomidae) in China under climate change. Insects.

[18] Calixto E.S., Paula-Moraes S.V. (2026). Tropical cyclones impact the dispersal of a globally invasive moth pest. Commun. Earth Environ..

[19] Guo Y., Zhao Z., Qiao H., Wang R., Wei H., Wang L., Gu W., Li X. (2020). Challenges and development trends of species distribution models. Adv. Earth Sci..

[20] Rotllan-Puig X., Traveset A. (2021). Determining the minimal background area for species distribution models: MinBAR package. Ecol. Model..

[21] Tessarolo G., Lobo J.M., Rangel T.F., Hortal J. (2021). High uncertainty in the effects of data characteristics on the performance of species distribution models. Ecol. Indic..

[22] Xu Z., Peng H., Peng S. (2015). Development and evaluation methods of species distribution models. Acta Ecol. Sin..

[23] Zhang Z., Jiang W., Qiu H., Xiao Z., Ling Z., Song J. (2026). Simulation of potential distribution of mangrove species in China and identification of restoration optimization sequences. Ocean Coast Manag..

[24] Luan S. (2022). Identification and Application of Suitable Habitats for the Himalayan Marmot in Yushu City. Master’s Thesis.

[25] Zhao G., Cui X., Sun J., Li T., Wang Q., Ye X., Fan B. (2021). Analysis of the distribution pattern of Chinese *Ziziphus jujuba* under climate change based on optimized biomod2 and MaxEnt models. Ecol. Indic..

[26] Thuiller W., Lafourcade B., Engler R., Araújo M.B. (2009). BIOMOD–a platform for ensemble forecasting of species distributions. Ecography.

[27] Huang Y., Dong Y., Huang W., Ren B., Deng Q., Shi Y., Bai J., Ren Y., Geng Y., Ma H. (2020). Overwintering distribution of fall armyworm (*Spodoptera frugiperda*) in Yunnan, China, and influencing environmental factors. Insects.

[28] Baloch M.N., Fan J., Haseeb M., Zhang R. (2020). Mapping potential distribution of *Spodoptera frugiperda* (Lepidoptera: Noctuidae) in Central Asia. Insects.

[29] Valavi R., Guillera-Arroita G., Lahoz-Monfort J.J., Elith J. (2022). Predictive performance of presence-only species distribution models:a benchmark study with reproducible code. Ecol. Monogr..

[30] Petrie R., Denvil S., Ames S., Levavasseur G., Fiore S., Allen C., Antonio F., Berger K., Bretonnière P.A., Cinquini L. (2021). Coordinating an operational data distribution network for CMIP6 data. Geosci. Model. Dev..

[31] Wen X., Zhao G., Cheng X., Chang G., Dong X., Lin X. (2022). Prediction of the potential distribution pattern of the great gerbil (*Rhombomys opimus*) under climate change based on ensemble modelling. Pest Manag. Sci..

[32] Xian X., Zhao H., Wang R., Huang H., Chen B., Zhang G., Liu W., Wan F. (2023). Climate change has increased the global threats posed by three ragweeds (*Ambrosia* L.) in the anthropocene. Sci. Total Environ..

[33] Yin C., Peng X., Huang X.L., Cai S.P., Song H.T. (2026). Prediction of potential suitable areas for 5 common *Coraebus* species (Coleoptera: Buprestidae) in China based on biomod2 combination model. Environ. Entomol..

[34] Guo K., Jiang X., Xu G. (2021). Potential suitable habitats of *Cyclobalanopsis lamellosa* and the effects of climate change on its distribution. Chin. J. Ecol..

[35] O’TOole M., Queiroz N., Humphries N.E., Sims D.W., Sequeira A.M.M. (2020). Quantifying effects of tracking data bias on species distribution models. Methods Ecol. Evol..

[36] Zhang Y., Li L., Zhang Y., Li J., Xue Y., Li D. (2019). Study on the effect of human disturbance on the connectivity and genetic diversity of Sichuan snub-nosed monkey (*Rhinopithecus roxellana*) in Shennongjia National Park. Acta Ecol. Sin..

[37] Elith J., Leathwick J.R. (2009). Species distribution models: Ecological explanation and prediction across space and time. Annu. Rev. Ecol. Evol. Syst..

[38] Xu J., Guan X., Huang Z., Yu C. (1999). Effects of different temperature and humidity combinations on growth, development and fecundity of *Spodoptera exigua*. Chin. J. Appl. Ecol..

[39] Huang L., Xue F., Chen C., Guo X., Tang J., Zhong L., He H. (2021). Effects of temperature on life-history traits of the newly invasive fall armyworm, *Spodoptera frugiperda* in Southeast China. Ecol. Evol..

[40] Zhang J., Yang Q., Zhao Z., Yu X., Wei J., Cheng H., Zhao X., Yang M., Jin B. (2024). The spatiotemporal patterns of the beet webworm (Lepidoptera: Crambidae) in China and possible dynamics under future climate scenarios. J. Insect Sci..

[41] Du Plessis H., Schlemmer M., Van den Berg J. (2020). The effect of temperature on the development of Spodoptera Frugiper-daSpodoptera frugiperda (Lepidoptera: Noctuidae). Insects.

[42] Zhang Z., Zheng Q., Zhang Y., Liu J., Yin X., Tang Q., Li J., Yuan Y., Li X., Zhu X. (2019). Determination of cold tolerance of laboratory populations of *Spodoptera frugiperda*. Plant Prot..

[43] Li X., Chen Z., Ba T., Yin X., Zhu X., Zhang Y. (2022). Effects of short-term high-temperature exposure on survival and reproduction of *Spodoptera frugiperda*. Plant Prot..

[44] Zhang X. (2022). Study on the Distribution of Suitable Areas, Generation Division and Migration Trajectory of *Spodoptera frugiperda* in Sichuan Province. Master’s Thesis.

[45] Zhao H. (2013). Study on the Fluctuation of China’s Corn Import and Export Trade. Doctoral Dissertation.

[46] Wang M., Huang W., Dong Y., Huang Y., Zhang B., Sun G., Elkahky M., Huang C. (2025). Temporal analyses of global suitability distribution for fall armyworm based on multiple factors. Ecol. Indic..

[47] Tay W.T., Meagher R.L., Czepak C., Groot A.T. (2022). Spodoptera frugiperdaSpodoptera frugiperda: Ecology, evolution, and management options of an invasive species. Annu. Rev. Entomol..

[48] Yu M. (2025). Study on the Occurrence, Spread and Phylogenetic Structure of *Cydia pomonella* in China. Master’s Thesis.

[49] Meng D. (2025). Analysis of Distribution Characteristics of Grassland Pests and Occurrence Trends of Major Pests in Hohhot. Master’s Thesis.

[50] Liu J., Zhu Y., Wu Q., Li Z., Rao Y., Hou S. (2020). Suitability analysis of Wheat streak mosaic virus in China based on CLIMEX and MaxEnt. Plant Quar..

[51] Urhausen S., Bradshaw C.D., Davie J., Eyre D., Hemming D., Li H., Taylor B., Zhang F. (2025). Climate-related risk to maize crops in China from fall armyworm, *Spodoptera frugiperda*. J. Pest Sci..

