# Supplementary material for: Simulation and Prediction of the Potential Distribution of Spodoptera frugiperda Under Current and Three Future Scenarios Based on the Biomod2 Model Under Global Climate Change
Source: Insects. 2026 Jul 27;17(8):772. doi: 10.3390/insects17080772 (PMC13513792; doi:10.3390/insects17080772)

Table S1. All environmental variables used in this study.

Abbreviation	Description
BIO1	Annual mean temperature(℃)
BIO2	Mean diurnal range (monthly mean (max temp- min temp))(°C)
BIO3	Isothermally (BIO2/BIO7)×100
BIO4	Temperature seasonality (standard deviation×100)(C of V)
BIO5	Max temperature of the warmest month(°C)
BIO6	Min temperature of the coldest month(°C)
BIO7	Temperature annual range(BIO5-BIO6)(°C)
BIO8	Mean temperature of the wettest quarter(°C)
BIO9	Mean temperature of the driest quarter(°C)
BIO10	Mean temperature of the warmest quarter(°C)
BIO11	Mean temperature of the coldest quarter(°C)
BIO12	Annual precipitation(mm)
BIO13	Precipitation of the wettest month(mm)
BIO14	Precipitation of the driest month(mm)
BIO15	Precipitation seasonality(C of V)
BIO16	Precipitation of the wettest quarter(mm)
BIO17	Precipitation of the driest quarter(mm)
BIO18	Precipitation of the warmest quarter(mm)
BIO19	Precipitation of the coldest quarter(mm)
gm_ve_v2	Percentage of vegetation coverage

Figure S1 Correlation heatmap of climatic variables.

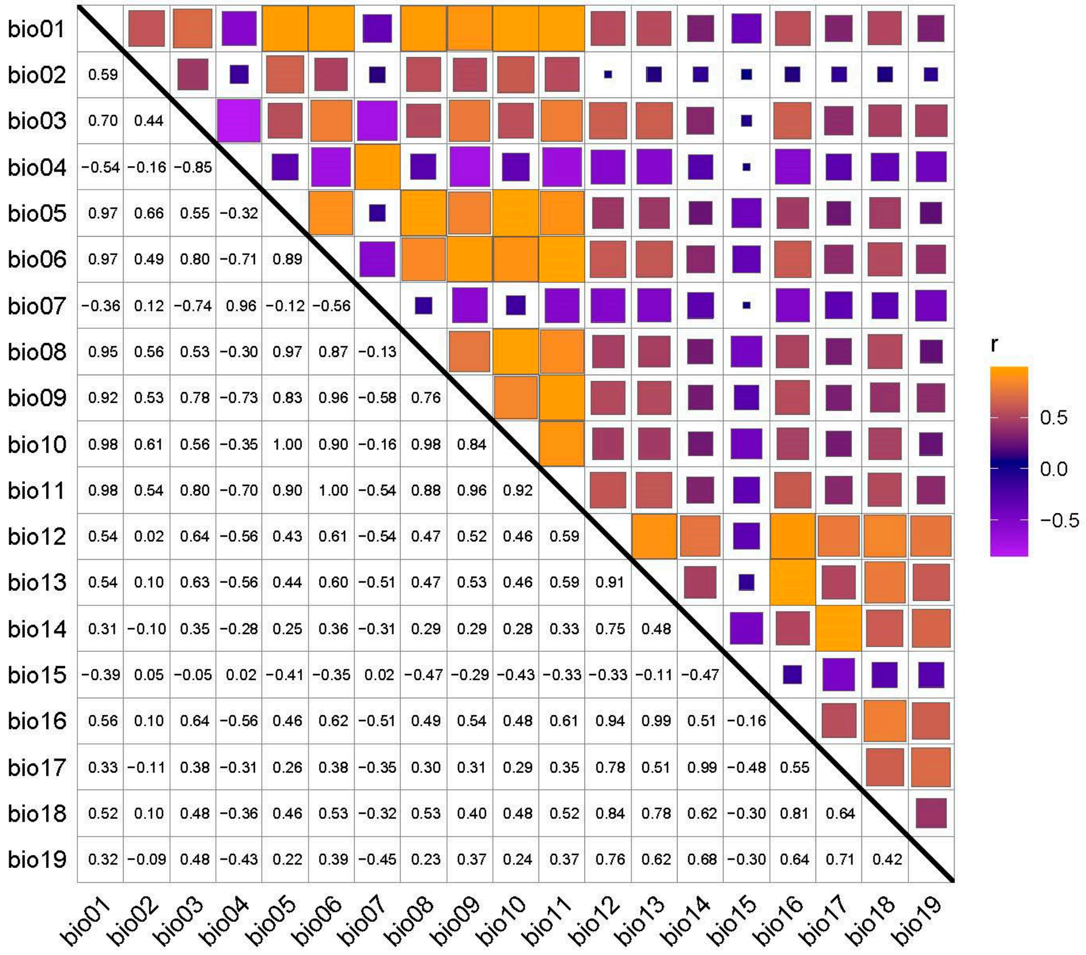

Supplement: Supplementary file 1 [file insects-17-00772-s001.zip › insects-4441486-supplementary.pdf]
